# Cherubism: A Case Report

**DOI:** 10.5005/jp-journals-10005-1019

**Published:** 2009-12-26

**Authors:** Virinder Goyal, Purshottam Jasuja

**Affiliations:** 1 Professor, Department of Pediatric Dentistry, Dasmesh Institute of Research and Dental Sciences, Faridkot, Punjab, India; 2 Reader, Surindera Dental College, Sri Ganganagar, Rajasthan, India

**Keywords:** Cherubism, multilocular radiolucencies, self limiting, autosomal dominant, fibro-osseous disorder, osteoclastic lesions, multinucleated giant cells.

## Abstract

Cherubism, a pediatric disease, is a self limiting non-neoplastic autosomal dominant fibro-osseous disorder of jaws. It is a self limiting disease and rarely apparent before the age of two years. It occurs in children and predominantly in boys. It is characterized by clinical bilateral swelling of cheeks due to bony enlargement of jaws that give the patient a typical ‘cherubic’ look. Regression occurs during puberty when the disease stabilizes after the growth period leaving some facial deformity and malocclusion. Cherubism may occur in solitary cases or in many members of the family, often in multiple generations. Radiographically, lesion appears as bilateral multilocular radiolucent areas. Since it was first described by Jones in 1933, many cases have been documented. Here a case of 8 years old cherubic child, with his clinical appearance as well as radiological evaluation and discussion about clinical outcome are presented. The patient was diagnosed but not treated.

## INTRODUCTION

Cherubism (OMIM#118400) is a benign, self limiting fibro osseous bone disease of childhood affecting only the jaws. It is evident around third or fourth year of life. In 1933, Jones^1^ first described the entity with its typical clinical features. Typically, the jaw lesions of cherubism are characterized by bilateral swelling of lower face. Because of this prominence of lower face, patient gives an appearance reminiscent of the ‘cherubs’ portrayed in Renaissance art, thus, this disease became known as Cherubism. It is one of the very few genetically determined osteoclastic lesions. Its gene^2^ is mapped on chromosome band 4p16.3 and is called^3^ SH3BP2 (for SH3 domain bind protein 2). The lesion tissue consists of vascular fibrous tissue containing varying numbers of multinucleated giant cells, which are diffused or focal. Radiologically, appearing as multilocular cystic lesion.

## CASE REPORT

**Fig. 1:**
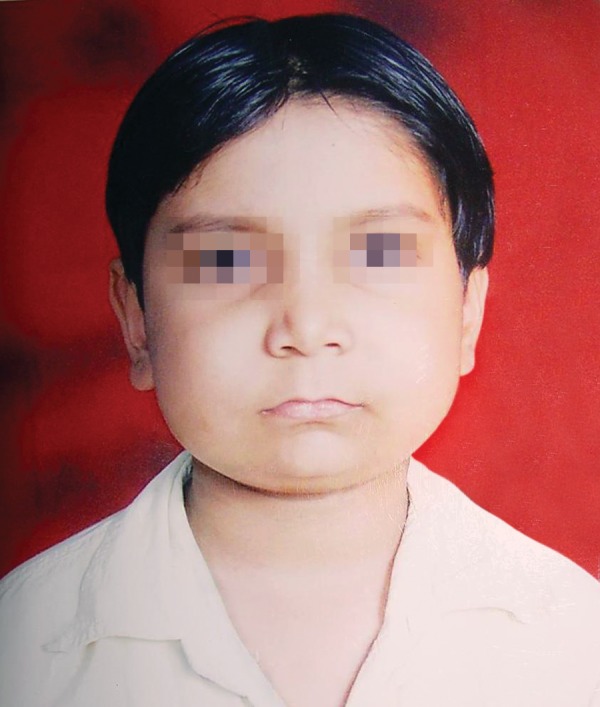
Case showing bilateral swellings of face

An 8 year-old male child (Fig. 1) reported to the Department of Pediatric Dentistry at Dasmesh Institute of Research and Dental Sciences, Faridkot, Punjab by his father for sole complaint of painless, progressive and bilateral enlargement of lower face and jaws.

The history revealed that the patient had been born as a full term normal delivery and showed no abnormalities until about the age of 2. But later symmetrical and bilateral swelling of lower face was seen. This enlargement had continued in gradually progressive fashion throughout the subsequent years. Family history showed that his father had a similar fullness of the cheeks in childhood that regressed after puberty. No siblings gave such a history.

On physical examination it was seen that patient was awell built, active and mentally alert. No abnormality was found on clinical examination of the chest, abdomen, cardiovascular and central nervous system. No cutaneous pigmentation or other congenital abnormality was present; there was no evidence of endocrinal disturbance.

Submandibular lymph nodes were bilaterally palpable , nontender and mobile.

On extraoral examination normal expression and color of the face was seen. There was no ophthalmic abnormality. There was symmetrical enlargement of both sides of mandible and minor involvement of maxilla. Enlargement was nontender and hard on palpation.

**Fig. 2:**
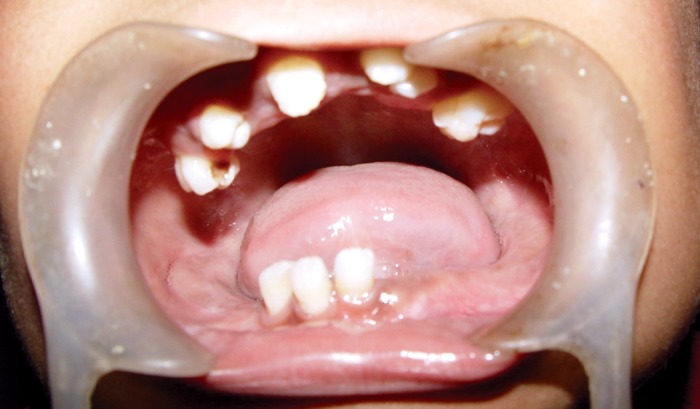
Intraoral view of case showing unerupted permanent and missing deciduous teeth

On intraoral evaluation few permanent teeth were seen with others missing (Fig. 2). Patient gave history of crowding in deciduous anterior teeth.

**Fig. 3:**
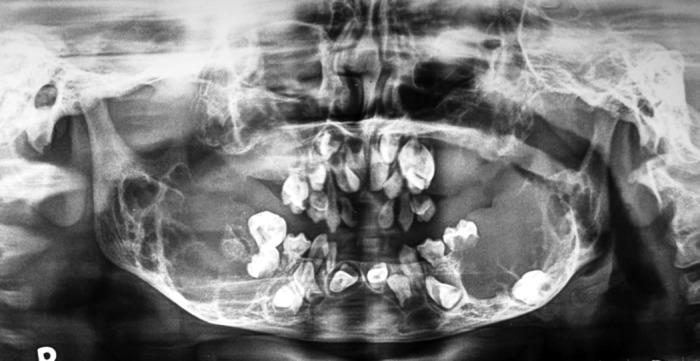
Radiograph showing multiple bilateral radiolucent areas extending upto base of condyles

Panoramic radiographs revealed extensive involvement of mandible. Multiple cystic areas were seen involving mandible on both sides extending up to the base of the condyle of the mandible (Fig. 3).

**Fig. 4:**
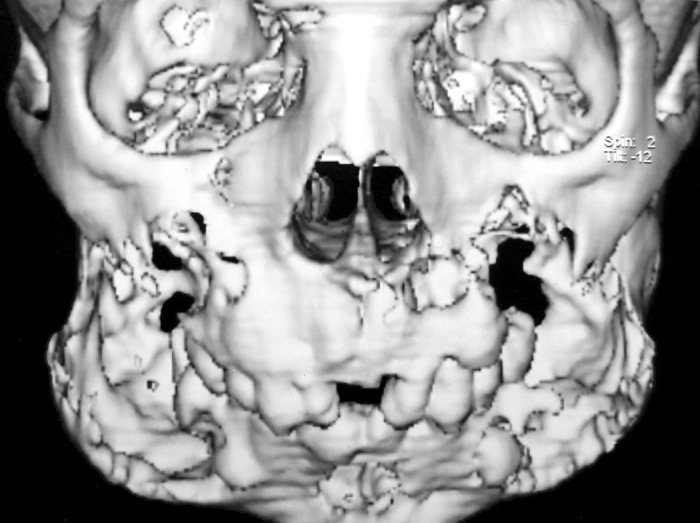
3D computed tomographic images showing osteolytic lesions in mandible and maxillae

**Fig. 5:**
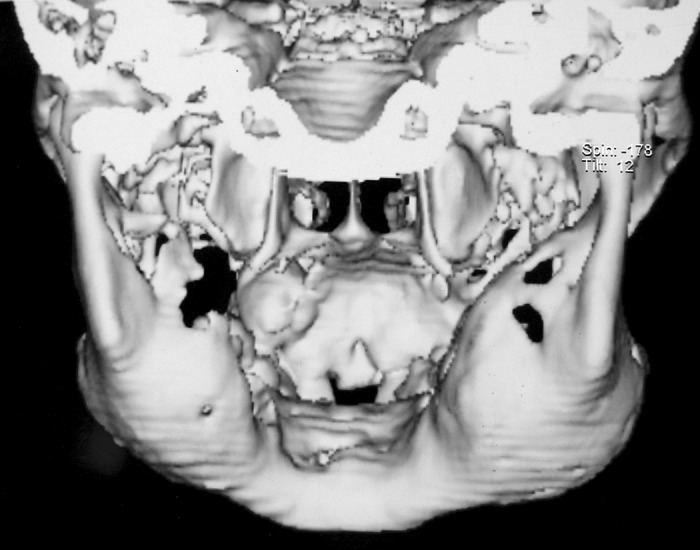
3D computed tomographic image of posterior view showing expansion of cortical plates with a normal base of skull

Computed tomography scan of facial bones was performed on subsecond multislice spiral CT scanner. 3 mm high resolution slices were obtained in the region of facial bones. 3D images were also reconstructed in the gray scale and color rendered mode. Slices revealed expansile osteolytic lesion involving body and ramus of the mandible on both sides. There was marked thinning of cortex on both sides. Extension of lesion was also seen in maxilla. Posterior walls of maxillary sinus appeared more affected than anterior walls (Fig. 4). Medial wall appeared normal on both sides. There is also expansion in the region of base of pterygoid plates on both sides. Visualized portion of base of the skull appeared normal (Fig. 5).

Laboratory investigations showed a hemoglobin level of 11.8 gm/dl (normal 13 to 18 gm/dl), hematocrit value of 38.4% (normal, 40 to 52%), each being slightly low, and an elevated alkaline phosphatase value of 623 IU/L (normal, 85 to 270 IU). Parathyroid hormone level and other lab investigations were within normal limits.

The lesion was diagnosed as grade II cherubism with the grading system^4^ for cherubism.

## DISCUSSION

Cherubism is a rare hereditary autosomal dominant benign lesion of childhood. It appears as bilateral painless swellings of mandible and maxilla which progress until puberty, and then spontaneously abates. In 1933^1^ Jones described the first case of cherubism in history of literature. To date, many cases have been added to the literature without restriction to any one country or ethnic group. Cherubism appears to be uncommon in India compared with the incidence in other countries. Here a case of 8 years old Indian cherubic child is reported.

According to World Health Organization (WHO)^5^ cherubism belongs to a group of nonneoplastic bone lesion that affect only the jaws. It is also considered member of the family of fibrous osseous diseases and some authors refer this disorder as familial fibrous dysplasia.

In 1978,^4^ Arnott suggested a grading system for the lesions of cherubism. Cherubism is divided into grades I, II, III and IV depending on location and the severity of involvement of jaws. These classifications are based on extent of lesion at the time of evaluation. The grade often increases on follow-up examination. Our case falls under grade II of this classification, i.e. involvement of both maxillary tuberosities as well as the mandibular ascending rami.

Clinical or radiographic findings of cherubism are not evident until the age of 14 months to three years of age. Typically, the earlier the lesion appears, the more rapidly it progresses. The progressive swelling of the face, with marked increase in fullness of cheeks and jaws, is common to all cases and is due to enlargement and expansion of the underlying bony structures, the skin and subcutaneous tissue being normal. The bilateral enlargement of maxilla when present, contributes to cherubic analogy by causing stretching of skin of the cheeks, thus exposing a thin line of sclera causing ‘eyes raised to heaven’ look. This was not reported in our case and is rarely encountered in other case reports. Frequently cherubism is accompanied by abnormalities in the configuration of dental arch and dental eruption. In severe cases tooth resorption occurs. The signs and symptoms of disease depend on the severity of the condition, range from clinically, radiologically undetectable features to grossly deformed jaws, upright palate, respiratory obstruction, impairment of vision and hearing. In few cases, cherubism has been described as being connected with other diseases and conditions such as Noonan’s syndrome. Jaw and face lesions with displaced teeth are the only clinical abnormalities present in the child reported here.

Radiologically, it is characterized by bilateral multilocular cystic expansion of the jaws. Cystic areas in the jaws become reossified resulting in irregular patchy sclerosis. The presence of numerous unerrupted teeth and the destruction of the alveolar bone may displace the teeth, producing an appearance referred as ‘floating tooth syndrome’. Classic ground glass appearance because of compressed trabecular pattern is seen but is nonspecific as in our case. In few cases radiographic examinations of other areas of the patient sometimes revealed peculiar cyst like changes in the ribs,^6^-^8^ humerus,^9^-^10^ femur and tibia.^11^

Computed tomography scan contributes to the diagnosis at all stages of cherubism. CT scan demonstrated that lesions were confined to the jaws and walls of maxillary sinus, whereas other facial bones were spared despite the severity in affected jaws. In our case CT revealed replacement of affected jaw bones by soft tissue density. There was marked thinning of cortex with break in the cortex on both sides. CT scan examination is useful in precisely demonstrating changes in lymphadenopathy and tissues surrounding cherubism.

Plain radiographs and computed tomography scans are sufficient for diagnosis of cherubism.

Magnetic resonance imaging, a noninvasive tomographic method is also useful to study the expansion of soft tissue, in particular in the aggressive forms and established preoperative vascular assessment.

On bone scintigrams, low radioactivity (cold areas) was sometimes observed in patients with jaw bone diseases. These scintigrams also represented characteristic findings of cherubism.

In general, cherubism has a good prognosis. Cherubism does not progress after puberty, and as the patient grows to adulthood, the entire jawbone lesion tends to develop a more normal configuration. Surgery is not a treatment of choice. But in case of expansion of tissue resulting in difficulty with airway or chewing capacity, biopsy and surgical intervention can be done. Medical attention for aesthetic and functional concern is required.

Cherubism can be substantially differentiated from fibrous dysplasia by the bilateralism of the cystic bone defect. It should also be differentiated from ameloblastoma, multiple dentigerous cyst, central giant cell granuloma and odontogenic myxoma. The only multifocal bone disease that could reasonably be expected to present as well-defined multiple jaw radiolucency and thus, diagnostic dilemmas is ‘nevoid basal cell carcinoma syndrome’. It does not however produce the facial swelling characteristic of cherubism.
